# Novel insights into sex-specific differences in heart rate variability and autonomic nervous system regulation during spawning behavior in chum salmon (*Oncorhynchus keta*) revealed by re-analysis of ECG logger data

**DOI:** 10.3389/fphys.2025.1511476

**Published:** 2025-05-29

**Authors:** Yuya Makiguchi, Takaaki K. Abe, Masaki Ichimura

**Affiliations:** ^1^ College of Bioresource Sciences, Nihon University, Fujisawa, Kanagawa, Japan; ^2^ Shibetsu Salmon Museum, Shibetsu, Japan

**Keywords:** salmonid reproduction, cardiovascular physiology, parasympathetic regulation, behavioral ecology, physiological adaptation, biologging, stress response

## Abstract

This study reanalysed electrocardiogram (ECG) data collected in a previous study on chum salmon to explore sex-specific differences in heart rate variability (HRV) and autonomic nervous system regulation during spawning. The prior research included six female and five male salmon with implanted ECG loggers, observed during spawning, and ten additional females for pharmacological experiments on autonomic nervous system effects. The analysis uncovered distinct HRV patterns between sexes. Females exhibited an increase in heart rate from 82.27 to 86.16 bpm post-spawning, while males decreased from 74.71 to 67.78 bpm. Breakpoint analysis identified four change points in female HRV and five in male HRV. Females displayed a heart rate decrease 21 min before spawning, while males maintained stable rates until spawning. Both sexes experienced cardiac arrest at spawning, consistent with the previous study. HRV changes did not always correspond directly with spawning behaviors, indicating autonomic nervous system involvement beyond physical exertion. Pharmacological experiments showed that atropine, a parasympathetic blocker, suppressed HRV and prevented cardiac arrest, emphasizing the key role of the parasympathetic system in regulating spawning-related HRV. The study suggests that HRV during salmon spawning is regulated by physical activity and autonomic nervous system control, with an important role in parasympathetic activation. This activation begins 20 min before spawning in females, serving as a preparatory mechanism for the physiological demands of spawning. These findings improve our understanding of salmonid reproductive physiology and may inform conservation strategies. Future research should investigate direct measurements of autonomic activity, environmental influences on HRV, and the relationship between HRV patterns and reproductive success. Combining HRV data with other physiological measurements could offer a more comprehensive understanding of the regulatory mechanisms underlying spawning behavior and the energetic costs associated with reproduction in salmonids.

## Introduction

The spawning period is a critical life event for salmonids, profoundly influencing individual survival and reproductive success. During migration from marine to freshwater habitats, salmon undergo complex physiological changes using considerable energy for swimming, sexual maturation, and spawning behaviors (Crossin et al., 2004; Quinn, 2018). For example, Chinook salmon reallocate a significant portion of their energy, with females dedicating approximately 14% to gonadal development and 78% to metabolism, whereas males allocate only 2% to gonadal development and 80% to metabolism (Bowerman et al., 2017). These adaptations are crucial for reproduction and are accompanied by significant shifts in gene expression to cope with environmental stressors such as changes in salinity and temperature (Evans et al., 2011; Miller et al., 2011).

Spawning behavior in salmonids reflects local adaptations, with traits like swimming performance are not necessarily directly linked to reproductive success (Quinn et al., 2001). The ability to optimize energy consumption and cardiovascular function during migration is necessary for Pacific salmon to meet the physical demands of upstream travel and prepare for spawning (Flores et al., 2012; Eliason and Farrell, 2016). However, salmon with limited energy reserves and high-stress indicators, such as elevated blood lactate, glucose, and cortisol levels, are at risk of premature mortality during migration (Cooke et al., 2006). Thus, understanding the physiological responses and behaviors during spawning is essential for understanding salmon’s reproductive ecology and population dynamics.

Heart rate variability (HRV) analysis has emerged as a valuable non-invasive tool to assess the interplay between fish’s sympathetic and parasympathetic branches of the autonomic nervous system (Vera and Priede, 1991; Altimiras et al., 1995). HRV measurements reveal how environmental factors such as water temperature influence cardiac function, reflecting physiological adjustments that maintain homeostasis. Notably, distinct HRV patterns during spawning have been observed; for example, male Atlantic salmon display higher activity levels and metabolic rates than females (Altimiras et al., 1996).

The autonomic nervous system is central to regulating HRV in fish by balancing sympathetic and parasympathetic inputs, with each branch contributing differently according to the physiological state (Cameron, 1979; Iversen et al., 2010). Pharmacological studies employing blockers such as atropine and propranolol have demonstrated that vagal (parasympathetic) regulation is critical for modulating heart rate and stroke volume, especially during rest, exercise, and stress. Sex-specific differences in autonomic regulation have been observed in spawning salmonids—males often exhibit higher heart rates and distinct low-frequency spectral peaks compared to females (Altimiras et al., 1996). Additionally, research on sockeye salmon suggests that females maintain higher resting heart rates due to lower cholinergic tone, implicating autonomic balance differences in spawning behavior and reproductive success (Sandblom et al., 2009). Despite these advances, the precise interactions between the sympathetic and parasympathetic systems under varying environmental stresses remain incomplete, highlighting the need for further investigation.

This study aims to clarify the relationship between heart rate variability (HRV) and spawning behavior in male and female chum salmon (*Oncorhynchus keta*), building on previous work by Makiguchi et al. (2009). Our primary objectives are to clarify the correlation between heart rate and specific spawning behaviors and to investigate the role of the autonomic nervous system during this critical life stage. By employing continuous HRV recordings via ECG loggers and pharmacological interventions, we seek to provide a detailed characterization of cardiac control under both physiological and environmental influences. Ultimately, our findings are expected to enhance understanding of reproductive physiology in salmonids and inform conservation and management strategies to improve salmon survival and reproductive success.

These studies emphasize the importance of high-resolution HRV analysis in elucidating the physiological mechanisms underlying salmon spawning. Our work provides insight regarding autonomic regulation during this critical life stage by integrating continuous ECG monitoring, detailed behavioral observations, and targeted pharmacological interventions. This comprehensive approach deepens our understanding of the interplay between cardiac function and spawning behavior. It lays the foundation for future research that may inform conservation and resource management strategies for sustainable salmon populations.

## Materials and methods

### Attachment procedure of ECG data logger

This study examined the dataset used by Makiguchi et al. (2009), which involved six female (mean ± s.d. fork length = 618 ± 22 mm; mean ± s.d. mass = 2648 ± 119 g) and five male (mean ± s.d. fork length = 655 ± 24 mm; mean ± s.d. mass = 3,028 ± 285 g) chum salmon (*Oncorhynchus keta*) implanted with ECG loggers (W400L-ECG, Little Leonard Co., Tokyo, Japan; 21 mm diameter, 110 mm length, 57 g mass in air). In addition to the ECG logger-implanted individuals, we used ten female chum salmon for pharmacological experiments. Specifically, three fish (mean ± s.d. fork length = 649 ± 32 mm; mean ± s.d. mass = 3,045 ± 65 g) were administered physiological saline as a control, three fish (mean ± s.d. fork length = 665 ± 50 mm; mean ± s.d. mass = 3,157 ± 82 g) were administered atropine, a parasympathetic nervous system inhibitor, and four fish (mean ± s.d. fork length = 624 ± 13 mm; mean ± s.d. mass = 2771 ± 20 g) were administered sotalol, a sympathetic nervous system inhibitor. The drugs were administered via a cannula inserted into the dorsal aorta of the individuals equipped with ECG loggers. For detailed information on the drug administration methods, please refer to Makiguchi et al. (2009). We anesthetized the fish and surgically sutured a copper disc bipolar electrode to the ventral side of each fish. The ECG loggers were attached to the dorsal side, anterior to the dorsal fin, and programmed to record at a sampling rate of 200 Hz. After a 24-hour recovery period, we placed the implanted fish in a spawning channel (3.8 m × 2.9 m × 1.1 m) at the Shibetsu Salmon Museum, Hokkaido, Japan. The channel was supplied with 16.8°C spring water and had a silt-free gravel bottom (Groot, 1996). We recorded the spawning behavior of the fish with a digital video camera and synchronized it with the ECG signals. Among the experimental data, we observed four pairs of male and female salmon for spawning behavior, with both individuals in each pair equipped with ECG loggers. Each of these four pairs exhibited two spawning events. Additionally, we paired two females with males without ECG loggers and obtained data only from the females in these pairs. These two pairs also exhibited two spawning events each. Furthermore, we paired one male with a female with no ECG logger attached and obtained data only from the male in this pair, with two spawning events observed. We observed 14 spawning events and collected electrocardiogram data associated with 22 spawning events from both males and females combined.

### Behavioral definitions

In this study, we defined two key spawning behaviors: redd digging and covering. Redd digging refers to the behavior where a female salmon uses her caudal fin to excavate gravel from the riverbed, creating a spawning nest. During this behavior, the female tilts her body and moves her caudal fin from side to side to displace gravel and form a depression for egg deposition. Covering behavior was defined as the action where a female uses her caudal fin to cover the deposited eggs with the excavated gravel. This action helps protect the eggs from predators and ensures a safe environment for hatching. Both behaviors were identified and analyzed using digital video recordings of the spawning events. We quantified the frequency of these behaviors by counting the number of instances per 10-minute interval.

### Data analysis

To examine the relationship between spawning behavior and heart rate, we set 2 h before and after the observed spawning time as the analysis interval. The 2-hour period before spawning corresponds to the timing when females determine the approximate location of the spawning bed and engage in digging behavior. We determined R-R intervals using ECGtoHR (Sakamoto et al., 2021), a computer program that calculates heart rate from electrocardiograms, running on IGOR Pro ver9.05 (WaveMetrics Inc., Lake Oswego, OR, United States). We used R software ver. 3.62 (Team, 2022) for the analysis. We set the significance level at 5%. When comparing heart rates before and after spawning in males and females separately, we used the lme4 library, specifying a Poisson distribution since the response variable, heart rate, is a count. Individual ID was treated as a random effect. Values are presented as mean ± standard deviation (s.d.). Heart rate is expressed as beats per minute (b.p.m.).

We also used the ‘strucchange’ library (Zeileis et al., 2003) in R software to investigate whether there were any breakpoints in the heart rate trends for both males and females during the period from 2 h before spawning to 2 h after spawning. The ‘strucchange’ library uses a function that detects structural changes in regression models, identifying potential breakpoints where the heart rate change significantly shifts along the time gradient. To determine the optimal number of breakpoints, we calculated the Bayesian Information Criterion (BIC) for each potential number of breakpoints. We selected the number of breakpoints resulting in the lowest BIC as the final number of change points (Zeileis et al., 2003). This method allows the most parsimonious model to be chosen, balancing the goodness of fit with the complexity of the model. This approach allows for identifying significant changes in heart rate trends over the spawning period, providing insights into the physiological responses of male and female salmon during this critical life stage.

## Results

**FIGURE 1:**
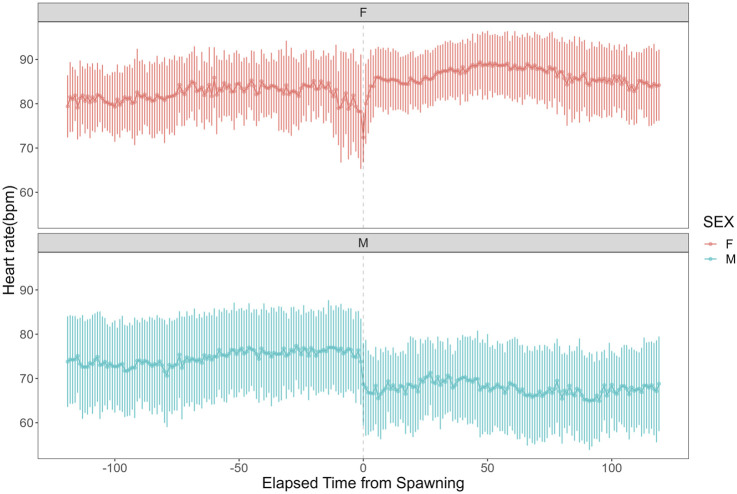
Heart rate time series data for female and male chum salmon (*Oncorhynchus keta*) during the 2 h before and after spawning. The x-axis represents time relative to spawning (in minutes), with 0 indicating the moment of spawning. Negative values denote time before spawning, while positive values indicate time after spawning. The y-axis shows the heart rate in beats per minute (bpm). The upper panel (pink) displays data for females (n = 6), while the lower panel (blue) shows data for males (n = 5). Each data point represents the mean heart rate; error bars indicate the standard deviation.

**FIGURE 2:**
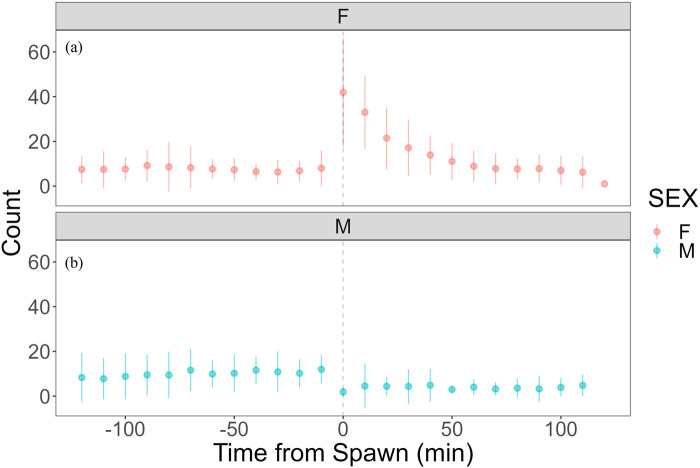
Time series data of female covering behavior and male quivering behavior in chum salmon (*Oncorhynchus keta*) during the 2 h before and after spawning. The x-axis represents time relative to spawning (in minutes), with 0 indicating the moment of spawning. Negative values denote time before spawning, while positive values indicate time after spawning. The y-axis shows the frequency of behaviors as counts per 10-minute interval. The upper panel (pink) displays covering behavior data for females (n = 6), while the lower panel (blue) shows quivering behavior data for males (n = 5). Each data point represents the mean count of behaviors per 10-minute interval, and error bars indicate standard deviation. Note the contrasting patterns between sexes: females show increased covering behavior immediately after spawning, while males exhibit peak quivering behavior just before spawning, followed by a sharp decline post-spawning. **(a)** Heart rate time series with identified change points. The x-axis represents time relative to spawning (in minutes), with 0 indicating the moment of spawning. The y-axis shows the heart rate in beats per minute (bpm). Vertical dashed lines indicate detected change points, with the numbers above showing the time (in minutes) relative to spawning when each change point occurred. Note the decrease in heart rate and increase in variability starting 21 min before spawning, followed by cardiac arrest at spawning (0 min), and subsequent increase until 46 min post-spawning. **(b)** Frequency of digging and covering behaviors with identified change points. The x-axis represents time relative to spawning (in minutes), with 0 indicating the moment of spawning. The y-axis shows the frequency of behaviors as counts per 10-minute interval. Vertical dashed lines indicate detected change points, with the numbers above showing the time (in minutes) relative to spawning when each change point occurred. Observe the decline in digging behavior until spawning, followed by a rapid increase in covering behavior immediately after spawning, with a slower rate of decrease starting 40 min post-spawning. These graphs illustrate the complex interplay between physiological and behavioral changes during the spawning process in female chum salmon.

**FIGURE 3:**
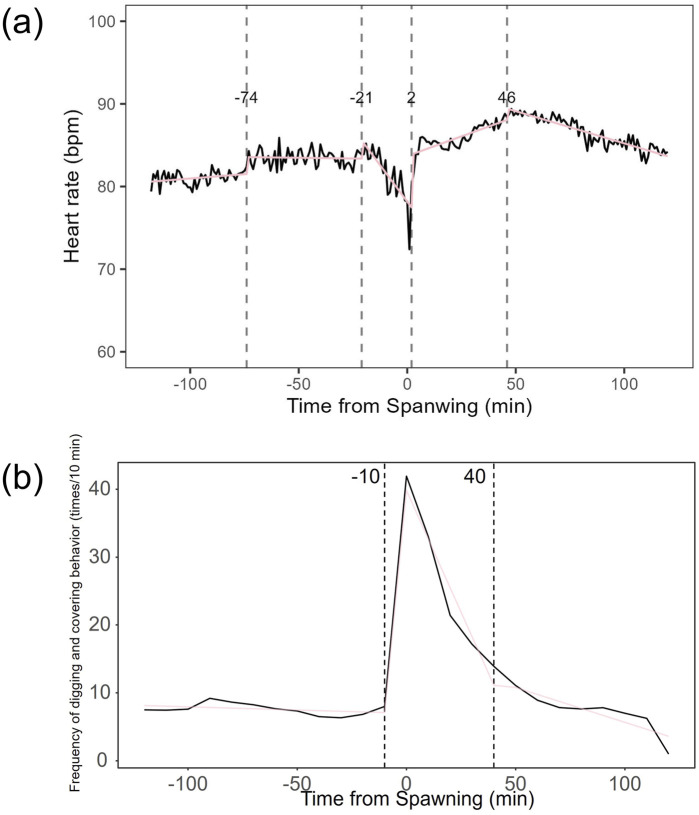
Breakpoint analysis of heart rate variability and behavioral changes in female chum salmon (Oncorhynchus keta) during spawning. **(a)** Heart rate time series with identified change points. The x-axis represents time relative to spawning (in minutes), with 0 indicating the moment of spawning. The y-axis shows the heart rate in beats per minute (bpm). Vertical dashed lines indicate detected change points, with the numbers above showing the time (in minutes) relative to spawning when each change point occurred. Note the decrease in heart rate and increase in variability starting 21 minutes before spawning, followed by cardiac arrest at spawning (0 minutes), and subsequent increase until 46 minutes post-spawning. **(b)** Frequency of digging and covering behaviors with identified change points. The x-axis represents time relative to spawning (in minutes), with 0 indicating the moment of spawning. The y-axis shows the frequency of behaviors as counts per 10-minute interval. Vertical dashed lines indicate detected change points, with the numbers above showing the time (in minutes) relative to spawning when each change point occurred. Observe the decline in digging behavior until spawning, followed by a rapid increase in covering behavior immediately after spawning, with a slower rate of decrease starting 40 minutes post-spawning. These graphs illustrate the complex interplay between physiological and behavioral changes during the spawning process in female chum salmon.

**FIGURE 4:**
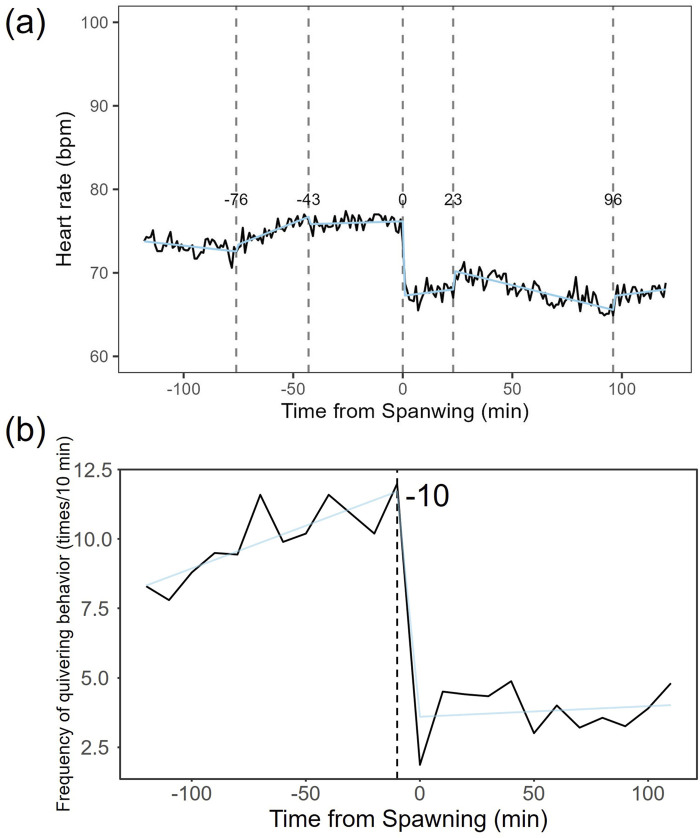
Breakpoint analysis of heart rate variability and courtship behavior changes in male chum salmon (*Oncorhynchus keta*) during spawning. **(a)** Heart rate time series with identified change points. The x-axis represents time relative to spawning (in minutes), with 0 indicating the moment of spawning. The y-axis shows the heart rate in beats per minute (bpm). Vertical dashed lines indicate detected change points, with the numbers above showing the time (in minutes) relative to spawning when each change point occurred. Note the initial decrease in heart rate from −120 to −90 min, followed by an increase until −43 min, stability until spawning, a sudden drop to 67 bpm at spawning (0 min) due to cardiac arrest, and sustained low heart rate with minor fluctuations post-spawning. **(b)** Frequency of quivering behavior with identified change point. The x-axis represents time relative to spawning (in minutes), with 0 indicating the moment of spawning. The y-axis shows the frequency of quivering behavior as counts per 10-minute interval. The vertical dashed line indicates the detected change point, with the number above showing the time (in minutes) relative to spawning when the change point occurred. Observe the increase in quivering behavior frequency from 2 h before spawning until spawning (ranging from 1.86 to 4.87 times/10 min), followed by a sharp decrease and sustained low levels post-spawning. These graphs illustrate the distinct patterns of physiological and behavioral changes in male chum salmon during spawning, highlighting the rapid shifts in both heart rate and courtship behavior around spawning.

Heart rate increased in females from 82.27 ± 7.59 bpm (n = 1,190) before spawning to 86.16 ± 7.18 bpm (n = 1,200) after spawning (p < 0.05). Conversely, heart rate in males significantly decreased from 74.71 ± 9.46 bpm (n = 1,190) before spawning to 67.78 ± 9.09 bpm (n = 1,200) after spawning (p < 0.05). Additionally, females exhibited significantly higher heart rates than males before and after spawning (p < 0.05). Figure 1 shows the heart rate time series data for both sexes during the 2 h before and after spawning. Figure 2 displays the time series data of male courtship behavior and female digging and covering behavior. Breakpoint analysis using the ‘strucchange’ library (Zeileis et al., 2003) in R software identified four change points in heart rate variability during spawning behavior in females (Figure 3a). Heart rate increased from 2 to 1.5 h before spawning and remained relatively stable until 21 min before spawning. At 21 min before spawning, heart rate decreased while heart rate variability increased, with a drop in heart rate observed at spawning due to cardiac arrest (Makiguchi et al., 2009). After spawning, the heart rate increased until 46 min post-spawning and then decreased until 2 h after spawning. The breakpoint analysis identified two change points in the frequency of female digging and covering behavior during spawning (Figure 3b). Female digging behavior declined until 2 h before spawning. Covering behavior occurred at spawning, increasing rapidly before decreasing. The rate of decrease slowed at 40 min after spawning, followed by a gradual decline. Changes in digging behavior frequency and heart rate did not clearly coincide before or after spawning. Female digging behavior occurred 6.3–9.2 times/10 min until spawning, with frequent covering behavior immediately after. Covering behavior frequency decreased after spawning, falling below 9 times/10 min 1–2 h post-spawning. In males, breakpoint analysis identified five change points for heart rate variability during spawning behavior (Figure 4a). Heart rate decreased from 2 h to 1.5 h before spawning, then increased until 43 min before spawning, after which it remained stable. During spawning, the heart rate drops to around 67 bpm due to cardiac arrest (Makiguchi et al., 2009). Heart rate remained low until 2 h after spawning, with only minor fluctuations. Breakpoint analysis detected one change point in male courtship behavior (Figure 4b). Courtship behavior frequency increased from 2 h before spawning until spawning, ranging between 1.86–4.87 times/10 min. After spawning, courtship behavior frequency decreased sharply and remained lower than pre-spawning levels, indicating that males rarely engage in courtship behavior after spawning.

**FIGURE 5:**
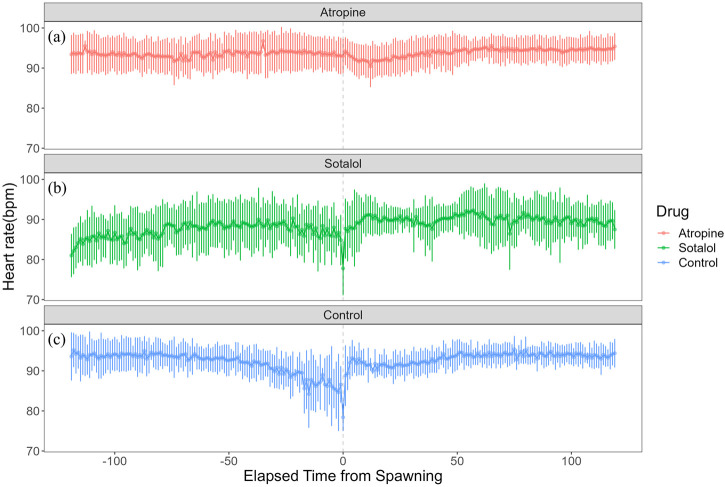
Effects of autonomic nervous system blockers on heart rate variability in female chum salmon (*Oncorhynchus keta*) during spawning. The x-axis represents time relative to spawning (in minutes), with 0 indicating the moment of spawning. The y-axis shows the heart rate in beats per minute (bpm). Each panel displays the heart rate time series for a different treatment group: Top panel (red line **(a)**): Atropine-treated group (parasympathetic blocker), Middle panel (green line **(b)**): Sotalol-treated group (sympathetic blocker), Bottom panel (blue line **(c)**): Sham control group.

To investigate the autonomic nervous system’s regulation of heart rate during spawning, we administered atropine and sotalol to females and compared their heart rate and variability with that of a sham control group. In the atropine-treated group, heart rate did not change from 93.5 ± 4.07 bpm (n = 579) before spawning to 93.9 ± 3.41 bpm (n = 595) after spawning. However, the sotalol-treated group showed an increase in heart rate from 87.2 ± 5.84 bpm (n = 828) before spawning to 89.8 ± 5.16 bpm (n = 825) after spawning (p < 0.05). The sham control group did not show changes in heart rate, with 92.0 ± 4.78 bpm (n = 592) before spawning and 93.0 ± 3.22 bpm (n = 599) after spawning. No significant differences in heart rate were found among the treatment groups before or after spawning. Figure 5 shows the heart rate time series for each treatment group. The atropine-treated group did not experience cardiac arrest, characterized by prolonged RR intervals, and showed decreased heart rate variability (Figure 5a). Conversely, the sotalol-treated group showed larger fluctuations in heart rate around spawning (Figure 5b). In contrast, the control group showed a sharp change in heart rate before and after spawning (Figure 5c).

**FIGURE 6:**
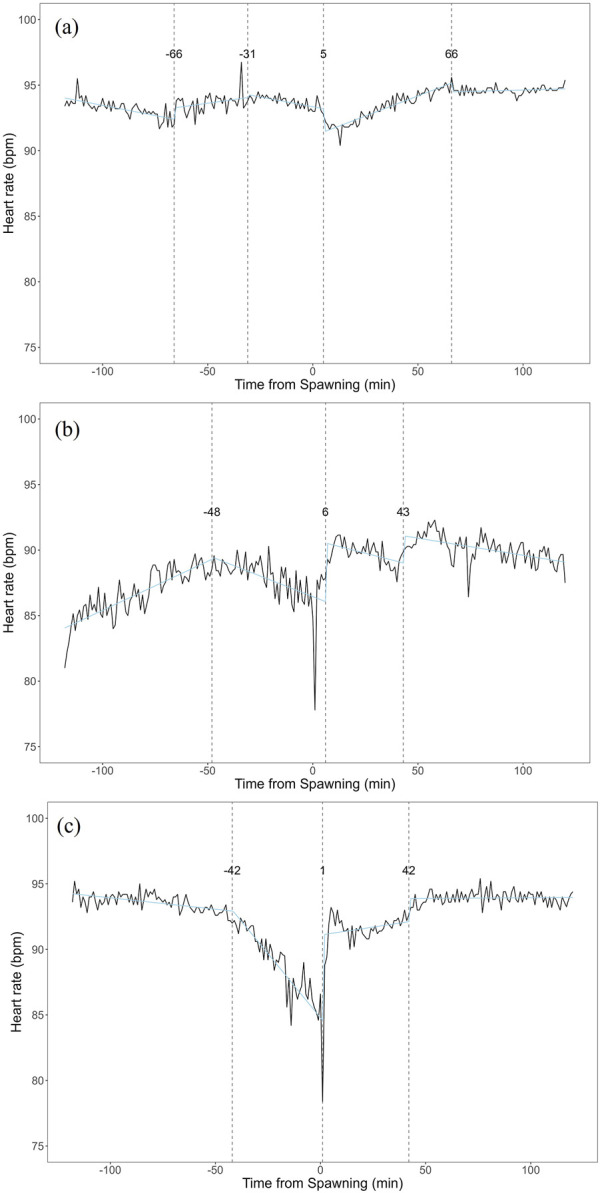
Breakpoint analysis of heart rate variability in female chum salmon (*Oncorhynchus keta*) under different autonomic nervous system blocker treatments during spawning. The x-axis represents time relative to spawning (in minutes), with 0 indicating the moment of spawning. The y-axis shows the heart rate in beats per minute (bpm). Vertical dashed lines indicate detected change points, with the numbers above showing the time (in minutes) relative to spawning when each change point occurred. **(a)** Atropine-treated group (parasympathetic blocker). Change points were detected at −66, −31, −5, and +66 min relative to spawning. **(b)** Sotalol-treated group (sympathetic blocker). Change points were identified at −48, +6, and +42 min relative to spawningc). **(c)** Sham control group. Change points were observed at −42, +1, and +42 min relative to spawning. Note that despite the different treatments, no clear distinctions in change point patterns were apparent among the groups. This suggests that factors beyond direct autonomic nervous system control may influence the overall temporal dynamics of heart rate variability during spawning.

Breakpoint analysis of heart rate variability revealed multiple change points in each treatment group (Figure 6). In the atropine-treated group, change points were identified at 66 min before, 31 min before, 5 min before, and 66 min after spawning (Figure 6a). The sotalol-treated group showed change points 48 min before spawning, 6 min after, and 42 min after spawning (Figure 6b). The sham control group had change points 42 min before spawning, 1 min after spawning, and 42 min after spawning (Figure 6c). No clear distinctions in change point patterns were apparent among the groups.

**FIGURE 7:**
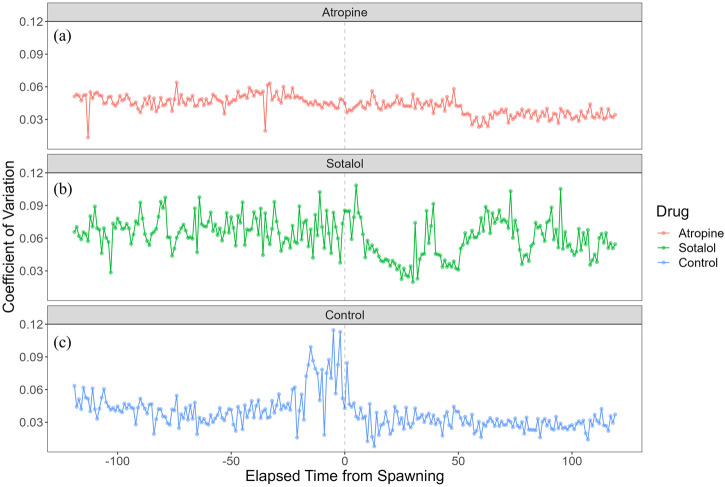
Coefficient of variation (CV) of heart rate in female chum salmon (*Oncorhynchus keta*) under different autonomic nervous system blocker treatments during spawning. The x-axis represents time relative to spawning (in minutes), with 0 indicating the moment of spawning. The y-axis shows the coefficient of heart rate variation (beats/minute). Each panel displays the CV time series for a different treatment group: **(a)** Atropine-treated group (red line): Note the consistently lower CV than other groups, indicating suppressed heart rate variability throughout spawning. **(b)** Sotalol-treated group (green line): Observe the more extensive CV around spawning and maintained high variability during most of the observation period, suggesting a more comprehensive range of heart rate changes. **(c)** Sham control group (blue line): Notice the sharp increase in CV 24 min before spawning, indicating that anticipation of spawning significantly affected heart rate variability. Post-spawning, the CV decreases from the large fluctuations observed pre-spawning. These graphs illustrate the differential effects of autonomic nervous system blockers on heart rate variability during the spawning process in female chum salmon.

Figure 7 shows each group’s coefficient of variation (CV) of heart rate during spawning. The atropine-treated group maintained a lower CV than the other two groups (Figure 7a). This suggests that atropine suppressed heart rate variability during the spawning process. The sotalol-treated group had a larger CV around spawning and maintained high variability during the observation period, except post-spawning (Figure 7b). This indicates a broader range of heart rate changes in the sotalol group. The control group showed a sharp increase in CV 24 min before spawning, suggesting that anticipating spawning significantly affected heart rate variability (Figure 7c). Post-spawning, the CV in the control group decreased from the large fluctuations observed pre-spawning.

## Discussion

### Sex-specific differences in heart rate variability patterns before and after spawning and their significance

This study analyzed the heart rate variability of salmon during spawning behavior in detail. A temporary increase in heart rate was observed immediately before spawning, followed by a rapid decrease in heart rate, or cardiac arrest, at the moment of spawning (Makiguchi et al., 2009). This cardiac arrest is suggested to be caused by increased parasympathetic nervous system activity (Taylor et al., 1999; Makiguchi et al., 2009). After recovery from cardiac arrest, the heart rate slowly recovers, but does not return to the pre-spawning level. This heart rate fluctuation is thought to reflect the energy expenditure and autonomic nervous system regulation during salmon spawning behavior. The increase in heart rate before spawning suggests increased sympathetic nervous system activity corresponding to the increased energy demand required for spawning (Altimiras et al., 1997; Taylor et al., 1999). On the other hand, the rapid decrease in heart rate during spawning is thought to be due to a rapid increase in parasympathetic nervous system activity to facilitate the release of eggs and sperm (Makiguchi et al., 2009). Such dynamic regulation of the autonomic nervous system is considered a physiological adaptation for fish to efficiently perform reproductive behavior (Nilsson, 1983). Furthermore, analysis of heart rate variability (HRV) suggested the possibility of changes in the autonomic balance of salmon during the spawning period. HRV is a method of evaluating autonomic nervous system activity using the variability of heart beat intervals as an index, reflecting the balance between the sympathetic and parasympathetic nervous systems (Akselrod et al., 1981; Altimiras, 1999). Changes in HRV during the spawning period may reflect the regulation of the autonomic nervous system in response to stress and changes in energy consumption associated with reproductive behavior (Campbell et al., 2004, 2006). However, in this study, specific HRV indicators (e.g., LF/HF ratio by frequency analysis) were not analyzed, and further investigation is needed to quantitatively evaluate changes in autonomic balance. In future studies, a more detailed analysis of HRV will allow a more detailed understanding of the dynamics of the autonomic nervous system in salmon during spawning behavior. In addition, it is necessary to observe heart rate variability in more detail under natural conditions by utilizing bio-logging technology to better understand the relationship between spawning behavior and heart rate variability (Warren-Myers et al., 2021).

### Relationship between heart rate variability and spawning behavior before and after spawning

The observed cardiac arrest during spawning suggests a potential adaptive strategy aimed at conserving energy and enhancing spawning efficiency. This phenomenon, also reported by Makiguchi et al. (2009) in chum salmon, may be common among salmonids. However, we did not observe a direct correspondence between the frequency of spawning behavior and HRV patterns. This discrepancy may be attributed to the complex nature of spawning, where HRV is also influenced by other environmental and individual factors. Additionally, sex differences in heart rate during the spawning period suggest potential variations in autonomic nervous system activity between males and females. Studies by Sandblom et al. (2009) indicate that the autonomic innervation of the heart differs between sexes in salmonids. The increase in heart rate after spawning in females compared to the decrease in males may reflect differences in the balance of sympathetic and parasympathetic activity. The results of the pharmacological experiments provide further support for these interpretations. The increase in heart rate after atropine administration suggests that parasympathetic activity is crucial for maintaining a lower heart rate during spawning. Furthermore, as Steele et al. (2011) have shown, β-adrenergic receptors play an important role in the autonomic control of the heart and these receptors may influence the changes observed in heart rate during spawning. These findings suggest that heart rate fluctuations in spawning salmon are controlled not by a single factor, but by a complex interaction of several autonomic and physiological factors.

### The role of the autonomic nervous system in heart rate variability during spawning

Pharmacological experiments using atropine and sotalol have provided key insights into the autonomic regulation of heart rate in salmon during spawning (Campbell et al., 2009; Bazmi and Escobar, 2022). Atropine, a muscarinic cholinergic receptor antagonist, increases heart rate by blocking the parasympathetic nervous system’s effects on the heart, removing inhibitory tone. Sotalol, a β-adrenergic receptor blocker, mainly antagonizes the sympathetic nervous system, reducing heart rate and contractility (Steele et al., 2011). These drugs are widely used in fish cardiac function studies to dissect the relative contributions of the parasympathetic and sympathetic systems. The use of atropine in particular highlights the strong vagal tone that suppresses heart rate in many fish species. For example, in Antarctic fish, pharmacological blockade of muscarinic receptors with atropine increased heart rate, demonstrating a significant cholinergic influence on resting heart rate (Campbell et al., 2009). Furthermore, the use of adrenergic antagonists, such as sotalol, allows the study of adrenergic contributions to cardiac function. Combining these pharmacological tools allows identification of the intrinsic heart rate of the cardiac pacemaker in the absence of autonomic input, providing a detailed understanding of the autonomic control of heart rate (Sandblom and Axelsson, 2011; Sandblom et al., 2016). In the study by Makiguchi et al., 2009, atropine administration did not prevent cardiac arrest, while sotalol administration did, and heart rate variability was similar to the control group. This suggests a key role for the parasympathetic nervous system in cardiac arrest during spawning. However, analysis of the coefficient of variation (CV) revealed that heart rate variability increased in the control group immediately before and after spawning, while this increase was blunted in the atropine and sotalol-administered groups (Watanabe-Asaka et al., 2022). This indicates that not only the parasympathetic, but also the sympathetic nervous system significantly affects heart rate variability during spawning. While the parasympathetic system appears to play a central role in the cardiac arrest itself, the sympathetic system also appears to be involved in the dynamic modulation of heart rate during the period from immediately before to immediately after spawning. These findings highlight the complex interplay of the autonomic nervous system in the control of heart rate during spawning, supporting previous knowledge about the importance of the parasympathetic nervous system, and suggesting a role for the sympathetic nervous system in regulating heart rate variability during spawning. This discussion emphasizes the importance of analyzing not only average heart rate but also the CV of heart rate variability to understand the dynamic regulatory actions of the autonomic nervous system.

### ECG loggers and future research

Advances in data logger technology have significantly improved our ability to monitor physiological parameters in fish over extended periods, enabling detailed studies of cardiac function during critical life stages. In our study, high-resolution ECG loggers allowed the identification of precise change points in heart rate variability (HRV) that correlate with distinct spawning behaviors in chum salmon. Rather than simply reiterating our results, our findings suggest that the pre-spawning decrease in HRV observed 21 min before spawning in females may represent a preparatory autonomic adjustment, likely mediated by increased parasympathetic activity. This anticipatory response, similar to what has been observed in other salmonids (Sandblom et al., 2009), appears to optimize cardiac performance in anticipation of the high energetic demands of spawning. Similarly, the rapid post-spawning drop in heart rate in males, coinciding with a decline in courtship behavior, indicates a swift autonomic modulation that may serve to conserve energy following intense activity. This rapid change highlights the dynamic nature of autonomic control during spawning (Makiguchi et al., 2009). Furthermore, our findings are consistent with the understanding that heart rate variability is not solely governed by physical exertion, suggesting complex autonomic nervous system responses during this critical life history event (Warren-Myers et al., 2021).

Importantly, our pharmacological experiments further support the role of parasympathetic control in these processes; the suppression of HRV by atropine treatment confirms that the autonomic nervous system is actively involved in regulating cardiac function during spawning (Makiguchi et al., 2009). In contrast, sotalol treatment did not prevent the occurrence of cardiac arrest, suggesting a more complex interplay between sympathetic and parasympathetic influences that merits further investigation. This finding aligns with other studies indicating that parasympathetic mechanisms are dominant in mediating cardiac arrest during spawning (Makiguchi et al., 2009) while also highlighting that the specific roles of sympathetic and parasympathetic systems can vary significantly across different physiological responses (Mann et al., 2010; Steele et al., 2011).

Looking ahead, future research should integrate ECG logging with additional physiological and environmental measurements—such as hormonal profiles, gastrointestinal blood flow, and energy expenditure—to elucidate the multi-faceted regulation of spawning behavior. This approach would allow for a deeper understanding of the interplay between internal physiological changes and behavioral responses during spawning (Brownscombe et al., 2019; Prystay et al., 2019). Comparative studies across salmonid species will also be essential to determine whether these autonomic responses represent a conserved adaptive mechanism or if they vary with ecological context. Such comparative research will help to assess the generality of the findings across different salmonid species and their ecological contexts (Campbell et al., 2009; Sandblom et al., 2009). Ultimately, a deeper understanding of these mechanisms will not only advance basic reproductive physiology in fish but may also inform conservation strategies and aquaculture practices by highlighting sex-specific responses to environmental stressors. Understanding sex-specific differences and responses is crucial for effective conservation efforts, as these differences can significantly impact mortality rates and reproductive success (Cooke, 2004; Sandblom et al., 2009). By focusing on the interpretation of our data and its implications, we aim to provide a comprehensive framework for future studies that bridges the gap between high-resolution physiological monitoring and practical applications in fish management and conservation. This interdisciplinary approach is crucial for developing effective strategies for sustainable fisheries management (Cooke et al., 2012; Sandblom et al., 2016).

## Data Availability

The raw data supporting the conclusions of this article will be made available by the authors, without undue reservation.
